# Patient and caregiver perspectives on transitioning to oral prophylaxis in the emerging hereditary angioedema treatment landscape

**DOI:** 10.1002/ccr3.5086

**Published:** 2021-11-16

**Authors:** Daniel F. Soteres, Fellicia Grimes

**Affiliations:** ^1^ Asthma and Allergy Associates Colorado Springs Colorado USA

**Keywords:** berotralstat, Haegarda, hereditary angioedema, shared decision‐making, switching, treatment burden

## Abstract

This report describes the successful transition of two familial patients with HAE from injectable to oral prophylaxis without tapering prior therapy or employing a complex transition protocol.

## 1 INTRODUCTION

In this study, we describe the experiences of a mother and daughter pair with HAE who transitioned from a long‐term injectable prophylactic treatment to oral berotralstat while enrolled in the APeX‐S (NCT03472040) study. Both patients safely transitioned to berotralstat monotherapy without tapering prior therapy or employing a complex transition protocol.

Hereditary angioedema (HAE) is a rare inherited disorder affecting an estimated 1 in 50,000 individuals worldwide.^1^, ^2^ It is characterized by recurrent, debilitating episodes of swelling in various parts of the body, such as the extremities, face, gastrointestinal (GI) system, and larynx (which can be life‐threatening).^3^, ^4^ Type 1 and type 2 HAE are caused by an inherited (~75% of cases) or sporadic (~25% of cases) deleterious mutation in the gene coding for the C1 esterase inhibitor (C1‐INH) protein, resulting in either a protein deficiency (type 1) or a dysfunctional protein (type 2).^3^, ^4^ C1‐INH is a serine protease inhibitor that plays an important role in regulating the kallikrein–bradykinin cascade involved in stimulating blood vessel permeability. When C1‐INH activity is reduced, bradykinin production increases, enhancing blood vessel permeability and triggering episodes of angioedema.^3^

There is no cure for HAE; therefore, therapeutic strategies focus on preventing (prophylactic) or treating (on‐demand) HAE attacks.^1^, ^4^ Many patients whose symptoms are not well controlled choose to receive long‐term prophylactic therapy supplemented by on‐demand treatment for breakthrough attacks.^1^, ^4^ Prophylactic therapy options have expanded rapidly in the past decade to now include two plasma‐derived C1‐INH concentrates, CINRYZE^®^ (C1‐INH) and HAEGARDA^®^ (subcutaneously delivered C1‐INH; C1‐INH‐SC), and two specific plasma kallikrein inhibitors, namely, TAKHZYRO™ (lanadelumab) and ORLADEYO™ (berotralstat).^5^, ^6^, ^7^, ^8^ Prior to these approvals, long‐term prophylactic HAE treatment options were restricted to attenuated androgens, which are associated with an adverse toxicity profile, and tranexamic acid, which has demonstrated limited efficacy as a preventive therapy.^1^

Advances in the treatment of HAE prophylaxis have greatly benefited patients by reducing symptoms and enabling self‐administration at home, which has increased patient satisfaction and quality of life.^6^, ^7^, ^9^, ^10^, ^11^ Despite these advances, some patients with HAE in the United States still experience a high burden of illness that affects their work and other activities.^12^ Many patients with HAE report having an informal caregiver, most commonly a family member with HAE, who assists in their HAE‐related medical care.^13^, ^14^ Patients with HAE can also experience burdens related to repetitive long‐term injectable treatments such as lanadelumab and C1‐INH‐SC, which are administered subcutaneously and can be burdensome, inconvenient, and associated with injection‐site reactions.^9^, ^10^, ^15^ In a 2018 report by the Center for Biologics Evaluation and Research and the U.S. Food and Drug Administration, patients with HAE considered the route of treatment administration to be an important factor in making treatment decisions, with oral preferred over subcutaneous (SC) administration and SC preferred over intravenous.^16^

Berotralstat, an orally available selective plasma kallikrein inhibitor for patients aged 12 years or older, provides patients with a safe and effective oral prophylactic option.^8^ In a 24‐week, double‐blind, placebo‐controlled phase 3 clinical trial (APeX‐2), the safety and efficacy of berotralstat (110 mg and 150 mg doses) were assessed in 121 patients aged ≥12 years old with type 1 or type 2 HAE.^17^ Both doses demonstrated a significant reduction in HAE attack rate compared with placebo; at week 24, the placebo group experienced an average of 2.35 attacks/month (baseline: 2.91 attacks/month), whereas the 110 mg group experienced an average of 1.65 attacks/month (*p *= 0.024; baseline: 2.97 attacks/month), and the 150 mg group experienced an average of 1.31 attacks/month (*p *< 0.001; baseline: 3.06 attacks/month).^17^ The most frequent adverse events (AEs) that occurred in ≥10% of patients in any treatment arm were upper respiratory tract infection, nausea, abdominal pain, vomiting, diarrhea, headache, and back pain, but no serious drug‐related AEs were observed in the study.^17^ The long‐term safety, effectiveness, and impact on the quality of life of oral berotralstat are being investigated further in an ongoing open‐label study in patients with type 1 or type 2 HAE who are ≥12 years of age (APeX‐S; NCT03472040).^18^ The primary endpoint of the study is safety, and the secondary endpoints of the study are efficacy and quality of life.^19^ Patients received open‐label berotralstat 110 mg or 150 mg; following the results from the APeX‐2 trial showing superior efficacy at 150 mg, patients on 110 mg were switched to the 150 mg dose. All data included in the following case reports are interim data from the APeX‐S trial.

With a new oral option available, some patients who are averse to scheduled injections may wish to switch from their long‐term injectable prophylactic medication to berotralstat. Currently, there is no consensus provided in the US HAE guidelines on how to transition patients from one long‐term prophylactic medication to another.^1^ Patients and physicians may have concerns about the safety and efficacy associated with switching prophylactic treatments, which may include concerns over possible worsening of symptoms and consequences of abruptly discontinuing one medication and initiating a new medication. It is important for patients to be involved in the decision to switch treatment and how and when the switch will occur to maximize safety and minimize patient anxiety.^1^ In this study, we report on two familial cases (daughter and mother) from the APeX‐S trial, describing patient and caregiver experiences with transitioning from previous SC prophylactic treatment to oral berotralstat monotherapy as a long‐term prophylactic therapy.

## 2 METHODS

This study conformed to the Declaration of Helsinki, and both patients provided informed consent prior to their inclusion in the study.

Two questionnaires, one patient and one caregiver, were developed by the authors regarding patient and caregiver experiences with prophylactic transition from C1‐INH‐SC to berotralstat. The patient questionnaire consisted of ten open‐ended questions, and the caregiver questionnaire consisted of three open‐ended questions. Interviews were conducted by the author Fellicia Grimes via web call a few months after the berotralstat transition. Open‐ended responses were provided by each patient/caregiver, and quotes from responses were used in the narrative provided in each case report.

### 2.1 Case 1: Daughter (adolescent patient)

Case 1 reports on a 13‐year‐old girl with type 1 HAE who was initially diagnosed at age 10. Her first prophylactic therapy was C1‐INH‐SC; 2,000 units injected subcutaneously every 4 days, which she received for 23 months prior to her entry in the APeX‐S study. Regular C1‐INH‐SC treatments worked well; in her words: “I barely had any attacks.” She decided to enroll in the APeX‐S trial “Because I would rather take a pill than take an injection twice a week.” After enrollment in the study, she initiated berotralstat therapy (150 mg once‐daily oral pill) while continuing her regular C1‐INH‐SC treatment schedule. Initially, she experienced mild GI discomfort related to berotralstat if she did not take the pill with a full meal. In her words: “If I forgot to take it at dinner and just took it with a snack, I would have a stomachache.” She did not take any medication to alleviate the discomfort, but she switched to consistently taking berotralstat with dinner, and her symptoms resolved. She continued to receive dual therapy (full doses of C1‐INH‐SC plus berotralstat) for ~4 months with no additional adverse effects or HAE attacks experienced.

She then decided to switch to single‐agent berotralstat because she “did not like taking injections, and a pill sounded easier and saved more time.” She noted that prior to the switch, “I was a little nervous, as I did not want to start swelling up again.” A smooth transition to oral berotralstat was achieved by discontinuing C1‐INH‐SC injections without tapering dose or reducing injection frequency. The patient did not report any challenges with this transition method.

The patient has received berotralstat monotherapy for ~3 months and experienced one HAE attack during that period. In her words: “I had an attack in my hand, but I am not sure what caused it.” The attack was treated with one dose of her usual on‐demand therapy, intravenous RUCONEST^®^ [C1‐INH (recombinant)], which resolved the symptoms within a few hours. Since transitioning to oral monotherapy, she described improvements to her quality of life: “I am doing great! I have not had any reactions and do prefer taking a pill to an injection.” and “When I was on [C1‐INH‐SC], I couldn't do extracurricular activities Wednesday nights and now I can.” A summary of treatment durations and HAE attacks experienced by Patient 1 is provided in Table 1.

**TABLE 1:** Number of attacks experienced during prophylactic treatment periods

	C1‐INH‐SC monotherapy	Dual therapy	Berotralstat monotherapy^a^
Patient 1			
Duration of treatment	23 months	4 months	3 months
Number of attacks	1	0	1
Patient 2			
Duration of treatment	9 months	3.5 months	4 months
Number of attacks	2	0	1

Abbreviation: C1‐INH‐SC, subcutaneously delivered C1 esterase inhibitor.

^a^ Berotralstat monotherapy still ongoing.

### 2.2 Case 2: Mother (patient and caregiver)

Case 2 reports on a 41‐year‐old woman with type 1 HAE who was initially diagnosed at age 14. She is the mother and primary caregiver of the adolescent patient in Case 1 and one other child with HAE (who was too young to enroll in the APeX‐S study at the time); thus, there are three individuals with HAE in her household. Her perspectives as a patient and a caregiver will be described in this section.

### 2.3 Patient perspective

As a patient, she previously received 6 months of weekly prophylactic intravenous injections of C1‐INH (recombinant). The injections were challenging; in her words: “my husband had to do it; I couldn't do it on myself, which was difficult.” She then switched to C1‐INH‐SC prophylaxis (5,000 units injected subcutaneously every 4 days), which she received for 9 months prior to enrolling in the APeX‐S trial. She experienced two HAE attacks during this 9‐month period. In describing the switch to C1‐INH‐SC, she said, “I don't know if it was necessarily better, but it was easier. I could give it to myself and it took a lot less time and a lot less prep.”

She decided to enroll in the APeX‐S trial because “I do not like taking injections. The process is cumbersome and requires more planning and time than a pill. With three of us needing medication and supplies, it takes up traveling space and time.” After enrollment, she initiated berotralstat therapy (150 mg once‐daily oral pill) while continuing her regular C1‐INH‐SC treatment schedule. She has regularly taken her pill with her largest meal of the day (dinner), but she occasionally experiences an “upset stomach, almost like indigestion” depending on the composition of the meal she consumes with her pill. In her words: “I don't notice it all the time; it depends on what I eat. If I tend to make poorer choices about what I eat, then it tends to be worse. It's not even just the medication doing it, it is more likely the diet.” She did not treat the discomfort with any medication. She received dual therapy (full doses of C1‐INH‐SC plus berotralstat) for ~3.5 months, which, in her words, “worked really well. I actually didn't experience any attacks at all while I was on the dual therapy.”

She decided to transition to berotralstat monotherapy because “I wanted to see if a pill would work on its own. I hadn't had attacks for some time, so I didn't mind taking a minor risk to see if a tablet would be enough [to control my symptoms].” She was “mildly concerned about having attacks return” if she switched to single‐agent therapy. The transition to the single‐agent berotralstat was achieved by immediate discontinuation of C1‐INH‐SC injections without tapering dose or reducing injection frequency, which did not present with any new challenges to the patient.

She has received berotralstat monotherapy for ~4 months and has experienced one HAE attack (abdominal) during that period, which responded well to her usual on‐demand therapy [(C1‐INH (recombinant)]. When describing the HAE attack, she said, “On demand worked the same; within 2–2.5 h, I was feeling better.”

Since transitioning to oral monotherapy, she described the impact on her quality of life: “I got some time back, and I don't have to worry about planning my injection around plans” and “It's much more convenient, much easier, and requires less preparation and planning.” In her words: “Most people, including myself, would much rather take a pill to control this, daily, than have to come up with twice‐a‐week injections.” A summary of treatment durations and HAE attacks experienced by Patient 2 is provided in Table 1.

### 2.4 Caregiver perspective

When describing her experiences as a caregiver prior to her daughter switching to berotralstat monotherapy, she noted, “It was a little stressful at times. If they did have an attack, we had to give intravenous injections, which is much harder to give in kids.” Also, she mentioned that “On [C1‐INH‐SC], we would all have to take injections together, so it was extremely time consuming. We made it part of our routine.” She also described travel constraints: “Any time we would have to travel … we had to take our rescue meds and [C1‐INH‐SC] with us.”

When describing her experiences as a caregiver after her daughter switched to berotralstat monotherapy, she reported, “It takes a lot less time. Even having one kid take the pill has been much easier.” She added, “It's made it easier to take care of normal life things, without having something get in the way. It's not even noticeable to take a pill; it takes two seconds as opposed to this huge ordeal of mixing medication, injecting it, etc.” She described reduced anxiety and stress as a caregiver: “It's less stress on everyone. It's a lot easier to make the decision to let her travel independently. I don't have to worry about her taking that medication on a plane or injecting it in a hotel room with a bunch of kids.”

She described changes in the quality of life of her daughter since switching to berotralstat monotherapy: “She loves it, she enjoys it; she has never liked needles.” She added, “She would much rather take a pill because it doesn't interfere with her other activities.”

## 3 DISCUSSION

In this study, we report on a mother and daughter with type 1 HAE who safely transitioned from C1‐INH‐SC to berotralstat prophylaxis. Both patients decided to switch to berotralstat treatment because they desired more personal time and freedom from injections. While in the APeX‐S trial, both patients overlapped therapies until they transitioned to berotralstat monotherapy (Table 1). It is important to note that this extended dual therapy period occurred during a clinical trial and is not medically necessary for transition to berotralstat in clinical practice; berotralstat reaches a steady state after 6–12 days of once‐daily dosing.^8^ No drug‐to‐drug interactions were observed for either patient during the dual therapy phase. Of note, C1‐INH‐SC treatments were not tapered prior to discontinuation and no changes in attack rate were observed during or immediately after the transition to berotralstat monotherapy. As of April 1, 2021, both patients remained on berotralstat monotherapy, experiencing only one attack each since transitioning (3‐ to 4‐month period); both attacks responded well to the usual on‐demand treatments of the patients.

After the initiation of berotralstat, both patients experienced occasional, mild abdominal discomfort when taking the capsule with certain foods (mother) or not enough food (daughter). These symptoms resolved for the mother when she chose healthier meal options and for the daughter when she took the capsule with a full meal (dinner). In the APeX‐2 study, the most common adverse events associated with berotralstat were GI related; however, these symptoms were generally mild and well tolerated and typically resolved on their own without the use of concomitant medication.^8^, ^20^

Initially, the patients had some concerns about the possibility of emergent attacks during the transition period, but the availability of on‐demand treatment eased these concerns. Both patients noted general improvements in quality of life after switching to oral berotralstat monotherapy, including increased independence from their disease and time gained from discontinuing their regular injections. The mother also described additional benefits as a caregiver, such as less stress, anxiety, and planning around the scheduled injections of her daughter.

Currently, there is a lack of consensus guidelines and related literature on the topic of HAE prophylactic transition, likely because most of the new therapies have only become available in the United States in recent years. The rapid expansion of the HAE prophylactic armamentarium has increased the complexity of prophylactic care, with new medications having different mechanisms of action, formulations, dosing schedules, and associated adverse effects. Therefore, making decisions about prophylactic HAE therapies requires effective communication between patient, physician, and caregiver to guide treatment decisions that consider all aspects of the treatment goals of the patient. Shared decision‐making and a personalized approach were taken with the patient treatment plans reported here. The treatment goals for both the patients and the physician were to optimize disease management and reduce the overall burden of disease and treatment.

In the absence of consensus guidelines, this case series provides two examples of patients who switched from C1‐INH‐SC to berotralstat safely. However, both patients transitioned to berotralstat in a clinical trial and their experiences may not be representative of the larger HAE population in clinical practice; therefore, we have not developed a detailed protocol for HAE prophylaxis transition in this report. Important considerations that contributed to the successful transition of both patients in this report include (1) shared decision‐making that included the goals and preferences of the patients before, during, and after transition, (2) scheduling the therapy transition during a time when the patient felt comfortable and was not experiencing high levels of stress, and (3) observing the patient closely during the transition period for any changes in HAE symptoms or adverse events related to the new prophylactic medication. Additional real‐world evidence is needed to develop a standard protocol for transitioning patients from one HAE prophylactic therapy to another in clinical practice.

In conclusion, this clinical report describes the successful transition of two patients with HAE in the same family from injectable prophylaxis to oral prophylaxis without tapering prior therapy or employing a complex transition protocol. Currently, there is no consensus or previous reports in the literature for transitioning patients with HAE from one prophylactic medication to another; however, in this case series, a mother and daughter transitioned to berotralstat after a dual therapy period and then discontinued C1‐INH‐SC without tapering. A limitation of this report is the small number of patient and caregiver examples described; more research studies are necessary to develop a specific protocol for the safe and effective transition of prophylactic HAE medications.

## CONFLICT OF INTEREST

Daniel F. Soteres, MD, MPH, is a clinical research investigator for Takeda and BioCryst and has accepted speaker or advisory board fees from Takeda, BioCryst, CSL Behring, and Pharming. Fellicia Grimes, BS, has no conflicts of interest to declare.

## AUTHOR CONTRIBUTIONS

Daniel F. Soteres served as treating physician, contributed to the development of questionnaires, and involved in the writing of the manuscript. Fellicia Grimes served as treating nurse, conducted patient interviews, and involved in the writing of the manuscript.

## ETHICAL APPROVAL

Institutional approval was not sought as the patient data were anonymized, and both patients provided written consent for their information to be published.

## PATIENT CONSENT STATEMENT

Both patients were informed that their anonymized information would be used for publication and provided their informed written consent prior to inclusion in the study.

## Data Availability

Data requests should be addressed to Daniel F. Soteres at DSoteres@aacos.com.
